# Association between Several Persistent Organic Pollutants and Thyroid Hormone Levels in Cord Blood Serum and Bloodspot of the Newborn Infants of Korea

**DOI:** 10.1371/journal.pone.0125213

**Published:** 2015-05-12

**Authors:** Sunmi Kim, Jeongim Park, Hai-Joong Kim, Jeong Jae Lee, Gyuyeon Choi, Sooran Choi, Sungjoo Kim, Su Young Kim, Hyo-Bang Moon, Sungkyoon Kim, Kyungho Choi

**Affiliations:** 1 School of Public Health, Seoul National University, Seoul, Republic of Korea; 2 College of Natural Sciences, Soonchunhyang University, Asan, Chungcheong, Republic of Korea; 3 College of Medicine, Korea University, Seoul, Republic of Korea; 4 College of Medicine, Soonchunhyang University, Seoul, Republic of Korea; 5 College of Medicine, Inha University, Incheon, Republic of Korea; 6 College of Medicine, Hallym University, Seoul, Republic of Korea; 7 College of Medicine, Jeju National University, Jeju, Republic of Korea; 8 College of Science and Technology, Hanyang University, Ansan, Gyeonggi, Republic of Korea; University of Cincinnati, UNITED STATES

## Abstract

Current knowledge on adverse endocrine disruption effects of persistent organic pollutants (POPs) among newborn infants is limited and often controversial. To investigate the associations between prenatal exposure to major POPs and thyroid hormone levels among newborn infants, both cord serum or maternal serum concentrations of polychlorinated biphenyls (PCBs), polybrominated diphenyl ethers (PBDEs), and organochlorine pesticides (OCPs) were compared with five thyroid hormones in cord serum of newborn infants as well as TSH in bloodspot collected at 2 day after birth (n=104). Since cord serum thyroid hormones could be affected by those of mothers, thyroid hormone concentrations of the matching mothers at delivery were adjusted. In cord serum, BDE-47, -99, and Σchlordane (CHD) showed significant positive associations with cord or bloodspot TSH. At the same time, *p*,*p'*-dichlorodiphenyldichloroethylene (*p*,*p'*-DDE) and hexachlorbenzene (HCB) showed negative associations with total T3 and total T4 in cord serum, respectively. Maternal exposure to *β*-hexachlorhexane (*β*-HCH), ΣCHD, ΣDDT, or *p*,*p'*-DDE were also associated with neonatal thyroid hormones. Although the sample size is small and the thyroid hormone levels of the subjects were within the reference range, our observation supports thyroid disrupting potential of several POPs among newborn infants, at the levels occurring in the general population. Considering the importance of thyroid hormones during gestation and early life stages, health implication of thyroid hormone effects by low level POPs exposure deserves further follow up investigations.

## Introduction

Persistent organic pollutants (POPs) have been detected in various environmental media and biota worldwide, even though many of these compounds including organochlorine pesticides (OCPs), and polychlorinated biphenyls (PCBs), had been banned for use several decades ago. Dichlorodiphenyltrichloroethane (DDT), one of best known OCPs, had been widely used for vector control, and has been frequently detected in humans worldwide [1, 2]. Polybrominated diphenyl ethers (PBDEs) are a group of emerging POPs that have been relatively recently recognized for widespread contamination and adverse health effects [3, 4]. In both wildlife and humans, adverse reproductive, developmental, neurologic, and endocrine health effects have been well-documented for many POPs [5].

POPs can cross the placenta during pregnancy [6], occur in breastmilk [7, 8], and therefore can affect the endocrine system of fetuses and breastfed infants. The early life stages are particularly vulnerable to chemical exposures because of incomplete metabolic activities [9], and rapid somatic growth and development [10]. The exposure to chemicals including POPs among developing fetus has been also suggested to be linked to the adverse health effects of later stages of life [11, 12].

Exposure to POPs has been associated with disruption of thyroid hormones in several human epidemiological studies [13–23]. Thyroid hormones are essential for normal growth and development of the fetus during the most of gestation period [24]. Among pregnant women, even moderate changes of thyroid hormone levels can be associated with adverse outcomes among the mother or her offspring [25–27]. In previous studies, while within reference range, higher maternal thyroid stimulating hormone (TSH) levels were associated with an increased risk of miscarriages, fetal and neonatal distress [28] and preterm delivery [29]. In addition, high free thyroxin (T4) levels within the normal reference range were associated with reduced preterm delivery rate [30]. Considering the importance of thyroid hormones among the developing fetus and newborn infants, therefore even small changes in thyroid hormones in these vulnerable populations are of potential concern. Associations between prenatal POPs exposure and thyroid hormone levels among newborn infants have been investigated by several groups, but the results are generally inconsistent and often controversial [16–18, 31–33]. Since thyroid hormone levels of cord blood serum are significantly influenced by maternal hormones [34, 35] and show significant fluctuations during and shortly after the delivery [36], it is quite challenging to identify the true association, if any, between POPs exposure and thyroid hormone levels in cord serum.

Our group has been investigating the POPs exposure among a matched pregnant woman and fetus panel of Korea, i.e., Children’s Health and Environmental Chemicals in Korea Panel (CHECK Panel) and their associations with adverse health outcomes including thyroid hormone levels [7, 37–39]. The CHECK Panel is composed of pregnant women-fetus pairs without any known occupational exposure pathways to major POPs such as OCPs, PCBs, and PBDEs, and was recruited since 2011 from four cities of Korea. Recently, from the pregnant women (n = 105) of this panel we reported negative associations between PBDEs/PCBs exposure and thyroid hormone levels [38].

In the present study, the associations between prenatal exposure to various POPs, and thyroid hormone status of the newborn infant population of the CHECK Panel were investigated. In order to account for the influence of maternal thyroid inputs, thyroid hormone levels in cord serum were adjusted for maternal thyroid hormone concentrations. For thyroid hormones, e.g., T4, significant maternal-fetal transfer during gestation has been reported [34]. Moreover, the levels of each of free T4, total T4, or total T3 were shown moderate associations between maternal and cord blood serum (Spearman’s rho = 0.13–0.24, *p*<0.05) in the mother-fetus pairs in the CHECK Panel (S1 Table). TSH levels measured at 2 days after birth were also employed, because of potential significant fluctuations in TSH levels during and shortly after the delivery [36]. The results of this study will help better understand the influences of prenatal POPs exposure on newborn infants, and identify the areas that warrant further investigations in the future.

## Methods

### Study population and sample collection

A total of 148 healthy pregnant women without histories of pre-pregnancy thyroid disease or pregnancy induced thyroid diseases were recruited before delivery from five university hospitals located in four cities of Korea, i.e., Seoul, Anyang, Ansan, and Jeju, reflecting a megacity, a mid-sized residence city, a mid-sized industrial city, and a mid-sized island city, respectively. Their matching fetuses were also recruited after full-term normal delivery. Details about the participating women can be found elsewhere [37]. During delivery, maternal blood and umbilical cord blood were collected, separated for serum on site, and stored in polypropylene cryovials at −70°C until analysis. Among them, POPs and thyroid hormones were analyzed in blood serum of 104 matching pairs (Table 1). In addition, on day 2 post-partum, bloodspot was obtained from each participating newborn infant by heel prick method, except for 5 infants who were sampled at days 5 and 7 post-partum. Institutional Review Boards of School of Public Health, Seoul National University, and all participating university hospitals approved the study, and the informed written consents were obtained from the participating women. All samples and data were processed blind.

**Table 1:** Characteristics of the study population.

Variable	n	Mean ± SD	Median	Range
**Maternal characteristics**				
Maternal age (years)	104	33.3 ± 3.9	33	25–46
Gestational age (days)	104	276 ± 7.6	276	261–293
BMI (kg/m^2^)	99	21.8 ± 21.1	21.1	15.6–33.6
Maternal weight gain during pregnancy (kg)	104	14.2 ± 13.7	13.7	3–32
Mode of delivery	104	NSVD^a^: 72 (69%), C-section^b^: 32 (31%)		
Parity	104	Primipara: 55 (53%), multipara: 49 (47%)		
Maternal serum hormones				
Free T3 (pg/mL)	104	2.53 ± 0.34	2.52	1.49–3.68
Total T3 (ng/mL)	104	1.44 ± 0.28	1.46	0.62–2.20
Free T4 (ng/dL)	104	0.92 ± 0.16	0.90	0.65–1.58
Total T4 (μg/mL)	104	9.18 ± 1.55	9.24	5.45–14.09
TSH (μlU/mL)	104	2.11 ± 1.20	1.87	0.01–5.61
**Infants characteristics**				
Infant sex	104	Female: 51 (49%), Male: 53 (51%)		
Birth weight (kg)	104	3.3 ± 0.4	3.3	2.5–4.3
Birth length (cm)	103	50.1 ± 1.9	50.0	45–54
Cord serum hormones				
Free T3 (pg/mL)	104	1.43 ± 0.23	1.39	1.00–2.39
Total T3 (ng/mL)	104	0.65 ± 0.11	0.63	0.48–1.08
Free T4 (ng/dL)	104	1.24 ± 0.11	1.24	0.94–1.52
Total T4 (μg/mL)	104	8.65 ± 1.19	8.61	5.65–11.59
TSH (μlU/mL)	104	10.27 ± 5.74	8.24	1.59–31.94
Bloodspot TSH^c^ (ulU/mL)	96	5.59 ± 3.08	5.05	0.10–15.90

^a^ Normal spontaneous vaginal delivery.

^b^ Caesarean section.

^c^ Bloodspot TSH was measured from bloodspot samples collected at day 2–7 post-partum. Most newborn babies were collected for bloodspot on day 2 (within 48 hrs) post-partum, but 3 and 2 infants were collected on day 5 and 7 post-partum, respectively.

### Data collection

Umbilical cord serum samples were processed and measured for 19 PCB congeners, 19 PBDE congeners, and 19 OCPs, consistently with previous study [38]. In addition, levels of five thyroid hormones, i.e., free/total triiodothyronine (T3), free/total T4, and TSH, were obtained. All thyroid hormones were analyzed by electrochemiluminescence immunoassay at Samkwang Medical Laboratories (Seoul, Korea), and the details for thyroid hormone measurements were provided in our previous report [38]. For POPs quantification, ^13^C-labeled 8 PCBs (EC9605-SS; Wellington Laboratories, Guelph, ON, Canada), ^13^C-labelled 6 PBDEs (MBDE-MXE; Wellington) and ^13^C-labeled 19 OCPs (ES-5349-L; Cambridge Isotope Laboratories, Andover, MA, USA) were used as surrogate internal standards. Limit of quantification (LOQ) was calculated as 10 times the signal to noise ratio. The respective LOQs for OCPs, PCBs and PBDEs were from 0.7 to 1.7 ng/g lipid weight (lw), from 0.8 to 8.3 ng/g lw and from 0.2 to 0.8 ng/g lw, respectively. All the POP concentrations were adjusted by the lipid contents of serum. Total lipid (mg/dL) was calculated from the concentrations of total cholesterol and triglyceride by the following equation [40]:

Totallipid=2.27*totalcholesterol+triglyceride+62.3

Total cholesterol and triglyceride were analyzed by enzymatic methods in a commercial clinical laboratory (Samkwang Laboratory, Seoul, Korea).

Details of sample preparation and instrumental analysis including quality assurance can be found in S1 Text. TSH levels from bloodspots (n = 96) were analyzed as a part of a national screening program of Korea, by radioimmunoassay. One-on-one interview with participating pregnant women was conducted at the time of enrollment. Demographic characteristics, physiological data, and pregnancy related record were obtained.

### Statistical analysis

Among 57 target POPs, eight compounds of which detection frequencies were ≥ 60% in serum and the sum of the isomers (ΣPCB, ΣPBDE, ΣDDT, Σchlordane (CHD), Σhexachlorhexane (HCH)) were employed for statistical analysis. For chemicals that were detected ≥ 75% of the population, a proxy value, i.e., limit of quantification (LOQ) divided by square root 2, was used to replace the non-detects [41]. For chemicals that were detected in < 75% but ≥ 60%, statistical analysis was conducted only with the detected values, in order to minimize the influence of non-detects.

Both dependent and independent variables were natural log-transformed to minimize the effect of highly right-skewed data, and were analyzed in multivariate analysis. To determine associations between POPs and thyroid hormone measurements in cord serum, multiple linear regression models were built with each chemical and each thyroid hormone. In addition, covariates that have been reportedly associated with the thyroid hormones elsewhere [42, 43] including age, maternal weight-gain during pregnancy, gestational age (days), mode of delivery, parity, pre-pregnancy BMI, and smoking status during pregnancy (yes/no) were included in the models. The former three covariates were continuous variables while the latter four were categorical. Because cord serum thyroid levels can be affected by the maternal input of thyroid hormones [34, 35], and also we found that the levels of free/total T4 and total T3 were positively correlated with its counterpart between maternal and cord serum in the CHECK population, therefore, respective thyroid hormone concentrations of the matching mother were also added into the model. For example, we included respective maternal thyroid hormone (e.g., free T4) as a covariate in the cord thyroid hormone (e.g., free T4) regression model. Unlike cord serum thyroid hormones, bloodspot TSH that was measured at 2 days of age was considered to be independent of maternal TSH input. As infant sex was determined to be significantly associated with bloodspot TSH, infant sex was added as covariate for analysis of bloodspot TSH, and smoking status was removed. For multivariate analysis, significance of linear model and normality of the residuals were checked. *P* < 0.05 was set as significant results, but considering small sample size, *p* < 0.10 was also shown as marginal significance. Percent changes in thyroid hormone levels expected for an interquartile range (IQR) increase of a given chemical concentration were back-calculated from the regression coefficients for each target chemical [44].

Sensitivity analysis was conducted if the following two criteria were met: 1) when two or more independent variables (i.e., POPs) were significantly associated with the same dependent variable (i.e., thyroid hormone levels); and 2) when the same independent variables were significantly correlated with each other based on the Spearman correlation test (S2 and S3 Tables). In the sensitivity analysis, the identified independent variables were then included in multivariate models to estimate *β*s and 95% confidence intervals (CIs) for thyroid hormone levels. SAS 9.3 (SAS Institute Inc., Cary, NC, USA) was used for statistical analyses.

## Results

### Characteristics of study population

General characteristics of the participating women and their newborn infants, along with the levels of thyroid hormones are summarized in Table 1. The ages of the participating pregnant women are generally in their early 30s, and about a half of the women were primiparae. Approximately two thirds of the participating women gave birth to babies by spontaneous vaginal delivery. Before pregnancy, most women were within normal weight range, with a mean pre-pregnancy BMI of 21.8 kg/m^2^. All TSH measurements in bloodspot papers were below 20 μlU/mL, showing no infant with congenital hypothyroidism [36, 45].

### POPs concentrations in cord blood serum

Several target compounds were detected in ≥ 60% of samples (Table 2). Among the target POPs, only *p*,*p’*-dichlorodiphenyldichloroethylene (*p*,*p’*-DDE) was detected in > 90% of cord serum samples. Detection levels of POPs in cord serum were generally similar to those of maternal serum, but detection frequencies were generally lower. The concentration of ΣPCB and ΣPBDE were higher in the cord serum than in the maternal serum. In cord serum, ΣPCB and ΣPBDE were detected at a median of 34.7 ng/g lw (interquartile range (IQR) of 18.4–55.5) and 8.8 ng/g lw (4.9–14.3), respectively, while those were detected at 23.5 (15.7–33.5) and 2.23 ng/g lw (1.5–4.6) in maternal serum. Most of the POPs were positively correlated with each other in cord or maternal serum. In cord serum, PCB-153 was correlated with many other chemicals including ΣPBDE, BDE-99, ΣHCH, *trans*-Nonachlordane (tNCHD), ΣDDT, *p*,*p’*-DDE (S2 Table). In maternal serum, *p*,*p’*-DDE was correlated with other chemicals like ΣPBDE, BDE-47, ΣHCH, *β*-HCH, ΣCHD, tNCHD, and ΣDDT (S3 Table).

**Table 2:** Cord blood serum concentrations of OCPs, PCBs, and PBDEs among the newborn infant population in Korea (n = 104).

	Cord blood serum				Maternal serum			
Chemical	Detection frequency		Median (IQR) ^a^		Detection frequency		Median (IQR)	
	n>LOQ	(%)	(ng/g lw)		n>LOQ	(%)	(ng/g lw)	
**ΣPCB**	97	**93.3**	34.7	(18.4–55.5)	96	**92.3**	23.5	(15.7–33.5)
**PCB-52**	66	**63.5**	5.4	(3.5–9.9)	69	**66.3**	1.0	(0.6–2.0)
**PCB-153**	78	**75.0**	10.5	(7.2–14.1)	95	**91.3**	8.4	(5.9–11.3)
**ΣPBDE**	88	**84.6**	8.8	(4.9–14.3)	97	**93.3**	2.2	(1.5–4.6)
**BDE-47**	77	**74.0**	3.0	(2.0–4.5)	92	**88.5**	1.2	(0.6–2.1)
**BDE-99**	67	**64.4**	3.0	(1.8–4.5)	29	**27.9**	0.7	(0.6–1)
**ΣHCH**	71	**68.3**	10.4	(7.8–13.9)	90	**86.5**	9.4	(6.0–12.9)
***β*-HCH**	70	**67.3**	7.5	(5.3–10.0)	88	**84.6**	7.5	(4.0–11.8)
**ΣDDT**	103	**99.0**	65.2	(46.3–97.2)	102	**98.1**	62.3	(42.6–81.3)
***p*,*p’*-DDE**	101	**97.1**	63.0	(44.0–91.5)	101	**97.1**	55.2	(38.7–73.9)
**ΣCHD**	82	**78.8**	2.6	(1.6–3.9)	96	**92.3**	3.9	(2.8–5.1)
**tNonaCHD**	70	**67.3**	1.8	(1.4–2.7)	92	**88.5**	2.1	(1.4–2.7)
**HCB**	69	**66.3**	12.7	(2.8–22.3)	80	**76.9**	5.5	(1.5–12.2)

^a^ Interquartile range (IQR) showing the 25^th^ and 75^th^ percentile values.

Only the compounds of which frequency of detection was greater than 60% in cord serum were shown. ΣPCB is the sum of all measured PCB congeners (PCB18, 28, 33, 44, 52, 70, 101, 105, 118, 128, 138, 153, 170, 180, 187, 194, 195, 199 and 206), and ΣPBDE is the sum of all measured PBDE congeners (BDE17, 28, 47, 49, 66, 71, 77, 85, 99, 100, 119, 126, 138, 153, 154, 156, 183, 184 and 191). ΣHCH included *α-*, *β-*, *γ-* and *δ*-HCH, ΣDDTs included *p*,*p′*-DDE, *o*,*p′*-DDE, *p*,*p′*-DDD, *o*,*p′*-DDD, *p*,*p′*-DDT and *o*,*p′*-DDT, and ΣCHDs included oxychlordane, trans-chlordane, cis-chlordane, transnonachlordane (tNonaCHD) and cis-nonachlordane.

### Association between cord serum POPs and thyroid hormone concentrations of newborn infants

Out of eight target POPs, five chemicals showed significant associations with thyroid hormone levels of newborn infants after adjustment of the covariates (Table 3). Significant negative association was detected between cord serum hexachlorobenzene (HCB) and total T4, with 5.8% (95% CI, -10.9 to -0.4) decline in total T4 by an IQR increase in HCB levels (increase from 2.8 to 2.3 ng/g lw, see Table 2 for this and other IQRs). Positive associations were detected between cord BDE-47 and bloodspot TSH, and between cord BDE-99 and cord TSH. An IQR increase of BDE-47 and -99 was associated with a 30.4% (95% CI, 2.6 to 65.7) increase of bloodspot TSH, and a 21.2% (95% CI, 0.1 to 46.8) increase of cord TSH, respectively. In addition, *p*,*p’*-DDE showed significant positive association with bloodspot TSH, and marginally significant negative association with total T3. A 16.5% (95% CI, 1.9 to 33.2) increase of bloodspot TSH was associated with an IQR increase of *p*,*p’*-DDE in cord serum. Cord ΣCHD and cord TSH levels were positively related as well. In contrast, ΣPCB and β-HCH concentrations showed negative trend with bloodspot TSH, but the association was not significant. The concentrations of PCB-52, -153, and tNCHD were not associated with thyroid hormones in cord blood serum samples of the present study.

**Table 3:** Associations between POPs concentrations and hormone levels in cord blood serum or in bloodspot of newborn infant population in Korea (n = 104).

		Cord blood											Bloodspot		
		Free T3 (pg/mL)		Total T3 (ng/mL)		Free T4 (ng/dL)		Total T4 (μg/dL)		TSH (μlU/mL)			TSH (μlU/mL)		
POPs (ng/g lw)		β	(95% CI)	β	(95% CI)	β	(95% CI)	β	(95% CI)	β	(95% CI)	n	β	(95% CI)	n
**PCB**															
	ΣPCB	0.018	(-0.02, 0.06)	**0.034^**	(0.00, 0.07)	-0.002	(-0.02, 0.02)	0.004	(-0.03, 0.04)	0.01	(-0.11, 0.13)	89	**-0.160^**	(-0.33, 0.01)	84
	PCB-52^+^	-0.03	(-0.08, 0.02)	0.002	(-0.05, 0.06)	-0.017	(-0.05, 0.02)	-0.028	(-0.08, 0.02)	0.04	(-0.13, 0.21)	62	0.121	(-0.09, 0.33)	58
	PCB-153^+^	0.019	(-0.06, 0.10)	0.022	(-0.05, 0.09)	0.021	(-0.02, 0.06)	-0.003	(-0.07, 0.06)	0.012	(-0.22, 0.24)	72	0.15	(-0.16, 0.46)	68
**PBDE**															
	ΣPBDE	0.024	(-0.02, 0.06)	0.023	(-0.01, 0.06)	0.002	(-0.02, 0.03)	0.011	(-0.03, 0.05)	0.072	(-0.06, 0.20)	81	-0.087	(-0.29, 0.12)	77
	BDE-47^+^	-0.014	(-0.09, 0.06)	-0.017	(-0.09, 0.06)	-0.015	(-0.06, 0.03)	-0.002	(-0.07, 0.06)	0.09	(-0.14, 0.32)	70	0.327*	(0.03, 0.62)	66
	BDE-99^+^	0.01	(-0.06, 0.08)	0.013	(-0.05, 0.08)	0.002	(-0.04, 0.04)	0.013	(-0.05, 0.07)	0.211*	(0.00, 0.42)	62	0.037	(-0.24, 0.32)	61
**OCP**															
	ΣHCH	-0.045	(-0.14, 0.05)	0.016	(-0.08, 0.11)	-0.041	(-0.10, 0.01)	-0.004	(-0.09, 0.09)	0.115	(-0.19, 0.42)	65	-0.057	(-0.38, 0.27)	64
	*β*-HCH^+^	-0.018	(-0.11, 0.07)	-0.002	(-0.09, 0.08)	-0.019	(-0.07, 0.03)	-0.016	(-0.10, 0.07)	0.129	(-0.14, 0.40)	64	**-0.270^**	(-0.57, 0.03)	63
	ΣCHD	0.021	(-0.03, 0.07)	0.029	(-0.02, 0.08)	0.005	(-0.02, 0.03)	0.021	(-0.03, 0.07)	0.162*	(0.01, 0.32)	76	0.197	(-0.05, 0.45)	73
	tNCHD^+^	0.011	(-0.06, 0.08)	0.029	(-0.04, 0.10)	0.012	(-0.02, 0.05)	0.031	(-0.03, 0.09)	0.117	(-0.08, 0.32)	65	-0.079	(-0.31, 0.16)	64
	ΣDDT	-0.048	(-0.12, 0.02)	-0.06	(-0.13, 0.01)	-0.007	(-0.05, 0.03)	0.017	(-0.05, 0.08)	0.069	(-0.16, 0.30)	95	0.11	(-0.24, 0.46)	90
	*p*,*p’*-DDE	-0.032	(-0.07, 0.01)	**-0.038^**	(-0.08, 0.00)	-0.002	(-0.02, 0.02)	-0.016	(-0.05, 0.02)	0.08	(-0.05, 0.21)	96	0.208*	(0.03, 0.39)	91
	HCB^+^	-0.018	(-0.05, 0.01)	-0.016	(-0.05, 0.02)	-0.004	(-0.02, 0.01)	-0.029*	(-0.06, 0.00)	0.062	(-0.03, 0.16)	64	-0.109	(-0.28, 0.06)	63

Signs * and ^ indicate statistical significance of regression parameter at p<0.05, and 0.1, respectively. All POPs concentrations and thyroid hormone levels were natural log—transformed. Results of association regarding free T3, total T3, free T4, total T4, and TSH were adjusted for age, gestation period, mode of delivery, parity, pre-pregnancy BMI, smoking status during pregnancy, and maternal weight gain during pregnancy. While infant sex was added as covariate and smoking status was removed for analysis of bloodspot TSH. Chemicals that were detected > = 75% of the population at concentrations greater than the limit of quantification, a proxy value of ‘limit of quantification divided by square root 2’ was used. For chemicals that were detected in <75% but > = 60%, statistical analysis was conducted with detected values only. Such chemicals are indicated by ‘+’.

Following the sensitivity analysis, most marginally significant associations between POPs and thyroid hormones disappeared. However, positive associations between BDE-47 and bloodspot TSH, and *p*,*p’*-DDE and bloodspot TSH were remained significant even after the sensitivity analysis (Table 4).

**Table 4:** Associations between serum POPs concentrations and thyroid hormones in the sensitivity analysis.

Cord POPs	Cord or bloodspot Thyroid hormones	β (95% CI)	n
ΣPCB	TT3	0.02 (-0.02, 0.06)	89
*p*,*p’*-DDE		-0.04^ (-0.09, 0.00)	
BDE-99	Cord TSH	0.22* (0.00, 0.42)	57
ΣCHD		0.13 (-0.06, 0.33)	
ΣPCB	Bloodspot TSH	0.07 (-0.13, 0.27)	64
BDE-47		0.38* (0.08, 0.67)	
ΣCHD	Bloodspot TSH	0.04 (-0.20, 0.28)	72
*p*,*p’*-DDE		0.54* (0.25, 0.82)	
**Maternal POPs**	**Cord or bloodspot Thyroid hormones**	**β (95% CI)**	**n**
*β*-HCH	fT3	-0.04^ (-0.08, 0.01)	94
*p*,*p’*-DDE		-0.02 (-0.07, 0.03)	
ΣCHD	fT4	-0.03 (-0.07, 0.01)	87
*p*,*p’*-DDE		-0.01 (-0.05, 0.02)	
BDE-47	Bloodspot TSH	0.02 (-0.13, 0.18)	88
ΣDDT		-0.13 (-0.58, 0.32)	
*p*,*p’*-DDE		0.45* (0.14, 0.76)	

Signs * and ^ indicate statistical significance of regression parameter (β) at p<0.05, and 0.1, respectively. ‘CI’ confidence interval; ‘fT3’ free T3; ‘TT3’ total T3; ‘fT4’ free T4. For the calculation of association, two or more independent variables that were determined as significant predictors to a given thyroid hormone, and at the same time were correlated each other, were added in the multiple regression analysis, in order to identify major predictors. In the regression model, interaction terms between the selected POPs were not included.

### Association between maternal serum POPs and thyroid hormone concentrations of newborn infants

Among target chemicals, maternal *β*-HCH, ΣCHD, ΣDDT, and *p*,*p’*-DDE were significantly associated with thyroid hormones of newborn infants. In addition, BDE-47, tNCHD, and HCB were marginally associated (Table 5). *β*-HCH showed negative associations with both free and total T3, and an IQR increase of *β*-HCH was related with 4.7% (95% CI, -8.4 to -0.7) decrease of free T3, and 4.1% (95% CI, -8.0 to -0.1) decrease of total T3. ΣCHD were negatively related with both free and total T4: 2.3% (95% CI, -4.4 to -0.1) decline of free T4 was associated with an IQR increase of ΣCHD in maternal serum. Both ΣDDT, and *p*,*p’*-DDE showed significant positive associations with bloodspot TSH (25% and 19% of increase following IQR increase of ΣDDT, and *p*,*p’*-DDE, respectively).

**Table 5:** Associations between POPs concentrations in maternal blood and thyroid hormone levels in cord blood serum or in bloodspot of newborn infant population in Korea (n = 104).

		Cord blood											Bloodspot		
		Free T3 (pg/mL)		Total T3 (ng/mL)		Free T4 (ng/dL)		Total T4 (μg/dL)		TSH (μlU/mL)			TSH (μlU/mL)		
POPs (ng/g lw)		β	(95% CI)	β	(95% CI)	β	(95% CI)	β	(95% CI)	β	(95% CI)	n	β	(95% CI)	n
**PCB**															
	ΣPCB	-0.028	(-0.08, 0.03)	-0.02	(-0.08, 0.03)	-0.004	(-0.04, 0.03)	0.005	(-0.06, 0.05)	-0.032	(-0.23, 0.16)	88	-0.157	(-0.43, 0.12)	84
	PCB-52^+^	-0.004	(-0.05, 0.04)	0.011	(-0.04, 0.06)	0.01	(-0.02, 0.04)	0.029	(-0.01, 0.07)	0.048	(-0.10, 0.20)	95	-0.027	(-0.24, 0.18)	91
	PCB-153	-0.011	(-0.05, 0.03)	-0.009	(-0.05, 0.03)	-0.004	(-0.03, 0.01)	0.011	(-0.03, 0.04)	-0.004	(-0.12, 0.13)	94	0.031	(-0.15, 0.21)	90
**PBDE**															
	ΣPBDE	-0.005	(-0.04, 0.03)	0.004	(-0.03, 0.04)	-0.002	(-0.02, 0.02)	0.013	(-0.02, 0.04)	-0.062	(-0.17, 0.05)	90	-0.005	(-0.17, 0.16)	86
	BDE-47	-0.005	(-0.04, 0.03)	-0.01	(-0.04, 0.02)	0.004	(-0.01, 0.02)	-0.008	(-0.03, 0.02)	-0.035	(-0.14, 0.07)	94	**0.130^**	(-0.01, 0.27)	90
**OCP**															
	ΣHCH	-0.021	(-0.06, 0.02)	-0.014	(-0.05, 0.02)	-0.005	(-0.03, 0.01)	0.002	(-0.03, 0.03)	0.057	(-0.06, 0.17)	83	-0.031	(-0.17, 0.11)	79
	*β*-HCH	-0.044*	(-0.08, -0.01)	-0.039*	(-0.08, 0.00)	-0.017	(-0.04, 0.00)	-0.028	(-0.06, 0.01)	0.067	(-0.06, 0.19)	95	0.041	(-0.14, 0.21)	91
	ΣCHD	-0.003	(-0.07, 0.07)	-0.013	(-0.08, 0.05)	-0.038*	(-0.08, -0.01)	**-0.056^**	(-0.12, 0.00)	0.006	(-0.22, 0.23)	88	-0.221	(-0.49, 0.05)	87
	tNCHD	-0.033	(-0.09, 0.02)	**-0.049^**	(-0.09, 0.01)	-0.015	(-0.04, 0.01)	-0.027	(-0.07, 0.02)	-0.01	(-0.18, 0.16)	95	0.048	(-0.22, 0.31)	91
	ΣDDT	-0.023	(-0.10, 0.05)	-0.023	(-0.10, 0.05)	-0.017	(-0.06, 0.02)	0.004	(-0.06, 0.07)	-0.035	(-0.22, 0.29)	94	0.345*	(0.00, 0.69)	90
	*p*,*p’*-DDE	**-0.035** ^**^**^	(-0.08, 0.01)	-0.028	(-0.07, 0.01)	**-0.020^**	(-0.04, 0.00)	**-0.034^**	(-0.07, 0.01)	-0.005	(-0.14, 0.13)	95	0.264*	(0.07, 0.45)	91
	HCB	-0.007	(-0.04, 0.03)	-0.015	(-0.05, 0.02)	0.001	(-0.02, 0.02)	-0.011	(-0.04, 0.02)	**0.105^**	(0.00, 0.21)	95	-0.095	(-0.25, 0.06)	91

Signs * and ^ indicate statistical significance of regression parameter at p<0.05, and 0.1, respectively. All POPs concentrations and thyroid hormone levels were natural log—transformed. Results of association regarding free T3, total T3, free T4, total T4, and TSH were adjusted for age, gestation period, mode of delivery, parity, pre-pregnancy BMI, smoking status during pregnancy, and maternal weight gain during pregnancy. While infant sex was added as covariate and smoking status was removed for analysis of bloodspot TSH. Chemicals that were detected > = 75% of the population at concentrations greater than the limit of quantification, a proxy value of ‘limit of quantification divided by square root 2’ was used. For chemicals that were detected in <75% but > = 60%, statistical analysis was conducted with detected values only. Such chemicals are indicated by ‘+’. Since BDE-99 were detected below 60% in maternal serum samples, they were not statistically analyzed.

In sensitivity analysis, *p*,*p’*-DDE was found to be a predominant determinant of bloodspot TSH (Table 4). Multivariate model analysis with bloodspot TSH and its three significant variables, i.e., BDE-47, ΣDDT and *p*,*p’*-DDE, showed even stronger association of *p*,*p’*-DDE (β = 0.45; 95% CI, 0.14, 0.76; *p*<0.01). However, both maternal *β*-HCH and ΣCHD became insignificant after adjustment of maternal *p*,*p’*-DDE in the free T3 and free/total T4 models.

## Discussion

### Influence of maternal thyroid hormones on the effect of POPs to neonatal thyroid hormones

Relatively few studies have been conducted on the association between POPs exposure and thyroid hormones among newborn infants (Table 6), and these observations generally showed inconsistent results. For example, while several studies reported the increase of TSH and decreases of T3 or T4, which are similar to the results of experimental studies on PBDEs [16, 21, 32, 46], PCBs [17, 22, 32, 47], or OCPs [20, 48, 49], the associations of the opposite directions or no association were also reported [18, 22, 33, 50–52], even within the same study [49]. Seemingly inconsistent associations between prenatal POPs exposure and the thyroid hormones in cord serum may be explained by several reasons. First, thyroid hormone levels in cord serum could be influenced by maternal transfer of thyroid hormones. Maternal thyroid hormones can be transferred to the fetus by perfusion system of placenta and blood exchange via cord. Fetuses depend entirely on the maternal supply of T4 during the first trimester and continue to depend on the maternal supply to varying degrees throughout the pregnancy [53]. At birth, around 30–60% of thyroid hormones in cord blood are of maternal origin [34, 35]. Therefore, thyroid hormones measured in cord serum could be influenced by the physiological or environmental factors that could affect maternal thyroid hormone levels. Second, several factors related to maternal, fetal, and delivery conditions may influence the thyroid status of the fetus. For example, delivery associated factors, such as mode of delivery, time of labor, emergency cesarean section, and induced labor, are reported to be related with intrapartum stress of mother and fetus [43], leading to rapid changes of thyroid hormone levels until shortly after birth [36]. Because controlling the labor- or delivery-related factors is almost impossible in human study, those variables that may influence the fetal thyroid hormones should be identified and adjusted in the study design.

**Table 6:** Associations between prenatal POPs concentrations in either maternal or cord blood and thyroid hormone levels of newborn infants.

Matrix	n	POPs	Thyroid hormone measurement						Reference
			fT3	TT3	fT4	TT4	TSH	matrix	
Cord serum	104	PCBs	-	-	-	-	- /-s	Cord serum & Bloodspot	This study
		**PBDEs**	-	-	-	-	**↑ / ↑s**		
		**OCPs**	-	-	-	**↓**	**↑ / ↑s**		
Cord serum	108	**PBDEs**			-		-	Cord serum	Kim et al. (2009b)
Cord serum	297	**PBDEs**			-	↓ /-s	-	Cord serum & Bloodspot	Herbstman et al. (2008b)
		**PCBs**			↓ /-s	- / ↓s	-		
Cord serum	39	**DDTs**			-	↓	-	Cord serum	Asawasinsopon et al. (2006)
Cord serum	92	**PCBs**	-			-	-	Cord blood	Takser et al. (2005)
Cord serum	9	**PBDEs**	-	-	-	-	-	Cord serum	Mazdai et al. (2003)
Cord serum	70	**HCB, PCBs, *p*,*p’-*DDE**					-s	Bloodspot	Ribas-Fito et al. (2003)
		***β*-HCH**					↑		
Cord plasma	410, 260	**PCBs**		-	-		-	Cord serum	Dallaire et al. (2008)
		**HCB**		-	↑		-		
Cord plasma	198	**PCBs, HCB**	↓		↓		-	Cord plasma	Maervoet et al. (2007)
		***p*,*p’-*DDE**			↓		-		
Cord blood	90	**PBDEs**	-	-	↓	-	-	Cord blood	Kim et al. (2011)
Cord blood	50	**PCBs**		-		↓	-	Cord blood	Zhang et al. (2010)
		**PBDEs**		-		-	-		
Cord blood	54	**PBDEs**	↓	↓	-	-	-	Cord blood	Lin et al. (2010)
**Maternal serum**	**104**	**PCBs**	-	-	-	-	**- /-s**	**Cord serum & Bloodspot**	**This study**
		**PBDEs**	-	-	-	-	**- /-s**		
		**OCPs**	**↓**	**↓**	**↓**	-	**- / ↑s**		
Maternal serum	79	**PCBs, OH-PCBs**			-s		↑s	Bloodspot	Hisada et al. (2014)
Maternal serum	260	**PBDEs**	-	-	↓	↓	-	Cord blood	Abdelouahab et al. (2013)
		**PCBs**	-	-	-	-	-		
Maternal serum	289	**PBDEs**					-	Cord serum	Chevrier et al. (2011)
Maternal serum	285	**PCBs**					↑s	Bloodspot	Chevirer et al. (2007)
Maternal blood	160	**PCBs**			-	-	-	Cord serum	Longnecker et al. (2000)

‘-’ no association;

‘↑’ positive association;

‘↓’ negative association (p<0.05);

Blank cell means data not available. ‘fT3’ free T3; ‘TT3’ total T3; ‘fT4’ free T4; ‘TT4’ total T4. In the present study, cord thyroid hormone levels were adjusted with maternal thyroid hormone levels in the model. Unless otherwise noted, all thyroid hormone measurements were from cord blood or cord serum. ‘s’ indicates the measurement in bloodspot of newborn infant.

To our knowledge, no study has adjusted maternal hormone effects for examination of the relation between POPs and thyroid hormones in cord serum. The reason for inconsistent directions of association between POPs exposure and thyroid hormone levels in cord blood serum among studies may be partly due to such fact. In the present study, in order to minimize the influences of the factors that would confound the association between cord serum thyroid hormone levels and prenatal POPs exposure, first, we included maternal thyroid hormone level as covariate in the multivariate analysis model. Second, TSH levels measured in bloodspot collected at 2 day post-partum were also employed for the association with prenatal POPs exposure. Since TSH is maintained consistent after 24 hrs of delivery, bloodspot TSH levels of the newborn infants at 2 day post-partum are considered relatively independent from the influence of the maternal thyroid hormone inputs [36]. In this context, Hardy et al. reported that bloodspot TSH showed better sensitivity than cord serum TSH, and was better than cord serum T4 for screening congenital hypothyroidism [54].

Unlike our expectation, however, adding maternal thyroid hormone levels as covariates did not generally influence the association between POPs and cord serum thyroid hormone levels, while the p values of the association became lesser for some POPs, e.g., ∑PCB in cord and tNCHD in maternal serum (S4 Table). Other important factors, rather than POPs exposure and maternal thyroid hormones, may exist, and may more accurately explain the thyroid hormone levels in cord serum. The utility of the adjustment of maternal thyroid hormones warrants further evaluation in other pregnant women-fetus pair populations.

### Association between POPs exposure and thyroid hormones

While statistical significance was not always detected, many POPs in cord serum generally showed negative associations with T3 or T4 levels, and at the same time showed positive associations with TSH levels in cord serum. Similar trends were also observed between POPs in maternal serum and thyroid hormones in cord serum. These observations are comparable to our recent observations in pregnant women [38] and several experimental studies [55–60], and suggest that at the current levels of exposure, several OCPs and PBDEs may be associated with subclinical hypothyroidism among pregnant women and newborn infants.

Our findings that prenatal exposure to OCPs (*p*,*p’*-DDE, HCB, β-HCH, and ∑CHD) appeared to be associated with T3, T4 of cord serum, and TSH of bloodspot were consistent with experimental study results [60–62]. In the 30-days exposure, reduced free and total T4 levels were observed following HCB treatment in rats [61, 62]. In experiment using sparrows, *p*,*p’*-DDT exposure led to decreased thyroid hormone levels, and inhibition of TSH receptor was suggested as one of the mechanisms for thyroid hormone disruption of DDTs [60, 63]. CHDs can interfere the cellular uptake of thyroid hormones, which may result in reduction of T3 or T4 levels [64].

The effect of OCPs appeared to be more evident among newborn infants compared to their matching mothers [38]. Early developmental stages are considered to be more sensitive to exposure to OCPs [65]. Potential epigenetic transgenerational action of several OCPs on endocrine system [66, 67] could also in part explain the observed sensitivity of newborn infants toward OCPs.

The significant positive associations between BDE-47 and bloodspot TSH, and between BDE-99 and cord serum TSH, which remained even after the adjustment of other related chemicals (Table 3 and S4 Table) suggest the effects of PBDEs on thyroid hormone homeostasis among newborn infants. PBDEs, especially BDE-47, were suggested to disrupt thyroid hormones through increase of hepatic enzyme related with glucuronidation, or decrease of transport protein such as transthyretin [68, 69]. Although human study is still sparse and the results are not consistent, our observation among the newborn infants is comparable to those of experimental studies [70, 71], and warrants further confirmation in the epidemiological studies.

The observation of no significant association between prenatal PCBs exposure and thyroid hormone levels among newborn infant population in the present study is different from the result in maternal serum where significant relationships between PCB exposure and total T3 or T4 were observed [38]. Total PCB in cord serum shows only weak effects (p < 0.10) on cord total T3 and bloodspot TSH levels (Table 3). However, these weak influences of total PCB disappeared by adding *p*,*p’*-DDE and BDE-47 of cord serum in the model (Table 4), suggesting that the effects of total PCB on thyroid hormones was probably confounded by PBDEs and OCPs. This null-association is not consistent with experimental results employing rats [58, 72, 73]. However, several epidemiological studies regarding thyroid hormone and PCB in newborns also failed to detect such significant observations [16, 31, 33, 49–51]. For example, no effect on thyroid hormones was observed with prenatal PCB exposure [33, 51], even though significant associations with other POPs were observed in the same population [16, 49, 50].

Why do PCBs influence thyroid hormone levels differently between pregnant women and newborn infants? While direct answer to this question is not ready, a couple of reasons can be considered in the future studies. First, potential differences in thyroid-related metabolic pathways between pregnant women and newborn infants should be considered [74]. In the same condition of marginal iodine deficiency, newborns were better protected from hypothyroxinemia compared to mothers, suggesting their different physiological responses [75]. Second, compared to other chemicals, PCBs are regarded to possess more complex mechanisms of action of thyroid-disruption, e.g., on the function of the TSH receptor, binding transport proteins, hormone receptor and gene expression, and excretion/clearance of thyroid hormones [76]. Such complex dynamics may have obscures true associations.

### Summary and implications

In the present study, the associations between thyroid hormone levels and prenatal POPs exposure were observed among the matching pregnant women-newborn infant pairs. Several OCPs, such as *β*-HCH, *p*,*p’*-DDE and HCB, and PBDEs, such as BDE-47 and BDE-99, were identified to be significantly associated with decreased T3 or T4, or increased TSH concentrations (Tables 3–5). However, the present study has a number of limitations that should be considered in the interpretation of the results. First, small sample size may lead to statistical significance by chance, and increased Type I error in regression models due to potential interactions between independent variables. Second, immunoassay measurements of free hormones can be affected by serum albumin, fatty acids, and other binding proteins that are reported to increase during pregnancy [77, 78]. This should be noted as a limitation in interpretation of the measurement data for free thyroid hormones. Third, thyroid hormones can fluctuate until shortly after the delivery, therefore the time of blood sample collection would influence the level of the hormones. While all the cord serum samples were collected immediately after the delivery, fluctuations in cord serum thyroid hormone levels cannot be ignored, and should be noted as another limitation.

However, our study is unique in that we have measured three groups of POPs (n = 57) and all five thyroid hormones in both maternal and fetal serum samples at the same time. In addition, sensitivity analysis was performed to confirm the influence of the predictors of thyroid hormone levels, i.e., POPs at the current levels of exposure. While the validation of our result in larger populations would be needed, the results of the present study provide another line of evidence that the current OCPs and PBDEs exposure among humans at sensitive life stages could influence the levels of thyroid hormones. The observed association between POPs exposure and bloodspot TSH of newborn infants also supports the same influence of the POPs toward thyroid hormone disruption among newborn infants. Even though thyroid hormone levels are within the reference range, small changes (< 25%) of maternal T4 or TSH during the early fetal period have been associated with adverse health outcomes [79]. Thus, considering the importance of thyroid hormones in rapidly developing bodies, public health implications of thyroid hormone disturbance among newborn infants should receive further investigations. Our observation also emphasizes the importance of further studies on implications at later life stages following the hormonal alteration occurring in the developing infants.

## Supporting Information

S1 Table Relationship between thyroid hormone concentrations in cord and maternal samples of the CHECK population. (DOCX)

S2 Table Spearman correlation table for detected POPs concentrations in cord serum. (DOCX)

S3 Table Spearman correlation table for detected POPs concentrations in maternal serum. (DOCX)

S4 Table Results of multivariate analysis with or without maternal thyroid hormone as covariates in the model. (DOCX)

S1 Text Supporting Materials and Methods. (DOCX)

S1 Dataset (XLS)
