# Supplementary material for: Association between Several Persistent Organic Pollutants and Thyroid Hormone Levels in Cord Blood Serum and Bloodspot of the Newborn Infants of Korea
Source: PLoS One. 2015 May 12;10(5):e0125213. doi: 10.1371/journal.pone.0125213 (PMC4429016; doi:10.1371/journal.pone.0125213)
Supplement: S1 Table — (DOCX) [file pone.0125213.s001.docx]

**Table S1. Relationship between thyroid hormone concentrations in cord and maternal samples of the CHECK population**

| **Spearman correlation matrix (n=258)** | |  | **Maternal serum thyroid hormones** | | | | |
| --- | --- | --- | --- | --- | --- | --- | --- |
|  |  |  | **Free T3** | **Total T3** | **Free T4** | **Total T4** | **TSH** |
| **Cord serum thyroid hormones** | **Free T3** | **ρ** | **0.051** | 0.022 | -0.050 | -0.004 | -0.101 |
|  |  | *p* | **0.417** | 0.724 | 0.426 | 0.943 | 0.104 |
|  | **Total T3** | **ρ** | 0.115^ | **0.128*** | -0.040 | -0.026 | -0.029 |
|  |  | *p* | 0.064 | 0.041 | 0.520 | 0.683 | 0.643 |
|  | **Free T4** | **ρ** | 0.050 | 0.067 | **0.210*** | 0.170* | -0.109^ |
|  |  | *p* | 0.421 | 0.281 | 0.001 | 0.006 | 0.079 |
|  | **Total T4** | **ρ** | 0.036 | 0.095 | 0.082 | **0.236*** | -0.114^ |
|  |  | *p* | 0.562 | 0.127 | 0.190 | 0.000 | 0.067 |
|  | **TSH** | **ρ** | -0.036 | -0.018 | -0.136* | -0.077 | **0.090** |
|  |  | *p* | 0.564 | 0.777 | 0.029 | 0.219 | **0.150** |

A total of 258 pairs of mother and newborn infant who participated in CHECK Panel were tested for the correlation test.

The p values showing statistical significance (p<0.05) are indicated by ‘*’, and those with marginal significance (p<0.1) are indicated by ‘^’.
