# Supplementary material for: Association between Several Persistent Organic Pollutants and Thyroid Hormone Levels in Cord Blood Serum and Bloodspot of the Newborn Infants of Korea
Source: PLoS One. 2015 May 12;10(5):e0125213. doi: 10.1371/journal.pone.0125213 (PMC4429016; doi:10.1371/journal.pone.0125213)
Supplement: S2 Table — (DOCX) [file pone.0125213.s002.docx]

Table S2. Spearman correlation table for detected POPs concentrations in cord serum

|  |  | **PCB-52** | **PCB-153** | **ΣPBDE** | **BDE-47** | **BDE-99** | **ΣHCH** | **β-HCH** | **ΣCHD** | **tNCHD** | **ΣDDT** | ***p,p’*-DDE** | **HCB** |
| --- | --- | --- | --- | --- | --- | --- | --- | --- | --- | --- | --- | --- | --- |
| **ΣPCB** | ρ | 0.429* | 0.294* | 0.170 | 0.296* | 0.212^ | 0.131 | -0.130 | 0.053 | 0.071 | -0.017 | -0.067 | 0.018 |
|  | p | 0.000 | 0.009 | 0.116 | 0.009 | 0.086 | 0.281 | 0.286 | 0.639 | 0.558 | 0.869 | 0.517 | 0.881 |
|  | n | 66 | 78 | 87 | 76 | 67 | 70 | 69 | 80 | 70 | 97 | 97 | 69 |
| **PCB-52** | ρ | 1 | 0.060 | 0.031 | 0.153 | 0.218 | 0.050 | -0.021 | -0.060 | -0.147 | 0.013 | 0.025 | 0.172 |
|  | p |  | 0.661 | 0.809 | 0.262 | 0.120 | 0.719 | 0.879 | 0.664 | 0.315 | 0.919 | 0.842 | 0.228 |
|  | n | 66 | 55 | 63 | 56 | 52 | 54 | 53 | 55 | 49 | 66 | 66 | 51 |
| **PCB-153** | ρ |  | 1 | 0.282* | 0.098 | 0.384* | 0.264* | 0.236^ | 0.219^ | 0.416* | 0.484* | 0.486* | 0.050 |
|  | p |  |  | 0.015 | 0.433 | 0.002 | 0.040 | 0.070 | 0.073 | 0.001 | <.0001 | <.0001 | 0.710 |
|  | n |  | 78 | 74 | 66 | 60 | 61 | 60 | 68 | 61 | 78 | 78 | 57 |
| **ΣPBDE** | ρ |  |  | 1 | 0.386* | 0.360* | -0.125 | -0.187 | 0.189 | 0.181 | 0.067 | 0.036 | 0.147 |
|  | p |  |  |  | 0.001 | 0.003 | 0.321 | 0.138 | 0.105 | 0.148 | 0.533 | 0.736 | 0.232 |
|  | n |  |  | 88 | 77 | 67 | 65 | 64 | 75 | 65 | 88 | 88 | 68 |
| **BDE-47** | ρ |  |  |  | 1 | 0.402* | 0.212 | 0.125 | 0.240^ | 0.070 | 0.122 | 0.146 | -0.023 |
|  | p |  |  |  |  | 0.002 | 0.113 | 0.359 | 0.052 | 0.603 | 0.290 | 0.206 | 0.861 |
|  | n |  |  |  | 77 | 59 | 57 | 56 | 66 | 57 | 77 | 77 | 58 |
| **BDE-99** | ρ |  |  |  |  | 1 | 0.257 | 0.184 | 0.383* | 0.215 | 0.213^ | 0.222^ | 0.185 |
|  | p |  |  |  |  |  | 0.060 | 0.187 | 0.002 | 0.106 | 0.083 | 0.071 | 0.169 |
|  | n |  |  |  |  | 67 | 54 | 53 | 62 | 58 | 67 | 67 | 57 |
| **ΣHCH** | ρ |  |  |  |  |  | 1 | 0.793* | 0.194 | 0.236^ | 0.198^ | 0.201^ | -0.001 |
|  | p |  |  |  |  |  |  | <.0001 | 0.126 | 0.074 | 0.099 | 0.093 | 0.992 |
|  | n |  |  |  |  |  | 71 | 70 | 64 | 58 | 71 | 71 | 52 |
| **β-HCH** | ρ |  |  |  |  |  |  | 1 | 0.176 | 0.277* | 0.366* | 0.386* | 0.082 |
|  | p |  |  |  |  |  |  |  | 0.167 | 0.037 | 0.002 | 0.001 | 0.568 |
|  | n |  |  |  |  |  |  | 70 | 63 | 57 | 70 | 70 | 51 |
| **ΣCHD** | ρ |  |  |  |  |  |  |  | 1 | 0.609* | 0.373* | 0.388* | 0.075 |
|  | p |  |  |  |  |  |  |  |  | <.0001 | 0.001 | 0.000 | 0.558 |
|  | n |  |  |  |  |  |  |  | 82 | 70 | 82 | 82 | 63 |
| **tNCHD** | ρ |  |  |  |  |  |  |  |  | 1 | 0.542* | 0.555* | 0.181 |
|  | p |  |  |  |  |  |  |  |  |  | <.0001 | <.0001 | 0.183 |
|  | n |  |  |  |  |  |  |  |  | 70 | 70 | 70 | 56 |
| **ΣDDT** | ρ |  |  |  |  |  |  |  |  |  | 1 | 0.978* | -0.051 |
|  | p |  |  |  |  |  |  |  |  |  |  | <.0001 | 0.679 |
|  | n |  |  |  |  |  |  |  |  |  | 103 | 103 | 69 |
| **p,p’-DDE** | ρ |  |  |  |  |  |  |  |  |  |  | 1 | -0.020 |
|  | p |  |  |  |  |  |  |  |  |  |  |  | 0.870 |
|  | n |  |  |  |  |  |  |  |  |  |  | 104 | 69 |

The p values showing statistical significance (p<0.05) are indicated by ‘*’, and those with marginal significance (p<0.1) are indicated by ‘^’.
