# Supplementary material for: Association between Several Persistent Organic Pollutants and Thyroid Hormone Levels in Cord Blood Serum and Bloodspot of the Newborn Infants of Korea
Source: PLoS One. 2015 May 12;10(5):e0125213. doi: 10.1371/journal.pone.0125213 (PMC4429016; doi:10.1371/journal.pone.0125213)
Supplement: S3 Table — (DOCX) [file pone.0125213.s003.docx]

Table S3. Spearman correlation table for detected POPs concentrations in maternal serum

|  |  | **PCB-52** | **PCB-153** | **ΣPBDE** | **BDE-47** | **ΣHCH** | **β-HCH** | **ΣCHD** | **tNCHD** | **ΣDDT** | ***p,p’*-DDE** | **HCB** |
| --- | --- | --- | --- | --- | --- | --- | --- | --- | --- | --- | --- | --- |
| **ΣPCB** | ρ | 0.245* | 0.691* | 0.322* | 0.079 | 0.072 | -0.008 | 0.047 | 0.135 | 0.024 | -0.034 | 0.208* |
|  | p | 0.016 | <.0001 | 0.002 | 0.449 | 0.515 | 0.938 | 0.664 | 0.190 | 0.820 | 0.745 | 0.042 |
|  | n | 96 | 96 | 90 | 95 | 84 | 96 | 89 | 96 | 95 | 96 | 96 |
| **PCB-52** | ρ |  | 0.229* | 0.021 | 0.244* | 0.305* | 0.179^ | 0.268* | 0.242* | 0.099 | 0.112 | 0.024 |
|  | p |  | 0.020 | 0.842 | 0.014 | 0.003 | 0.070 | 0.008 | 0.014 | 0.321 | 0.261 | 0.808 |
|  | n |  | 102 | 97 | 102 | 90 | 103 | 96 | 103 | 102 | 103 | 103 |
| **PCB-153** | ρ |  |  | 0.053 | 0.031 | 0.143 | 0.082 | 0.062 | 0.145 | 0.075 | 0.016 | 0.193* |
|  | p |  |  | 0.605 | 0.760 | 0.181 | 0.415 | 0.551 | 0.147 | 0.456 | 0.870 | 0.053 |
|  | n |  |  | 96 | 101 | 89 | 102 | 95 | 102 | 101 | 102 | 102 |
| **ΣPBDE** | ρ |  |  |  | 0.451* | -0.034 | -0.139 | 0.027 | 0.057 | 0.222* | 0.217* | 0.355* |
|  | p |  |  |  | <.0001 | 0.758 | 0.175 | 0.800 | 0.578 | 0.030 | 0.032 | 0.000 |
|  | n |  |  |  | 97 | 85 | 97 | 91 | 97 | 96 | 97 | 97 |
| **BDE-47** | ρ |  |  |  |  | -0.039 | -0.121 | 0.098 | 0.103 | 0.292* | 0.287* | 0.242* |
|  | p |  |  |  |  | 0.714 | 0.227 | 0.344 | 0.301 | 0.003 | 0.003 | 0.014 |
|  | n |  |  |  |  | 90 | 102 | 96 | 102 | 101 | 102 | 102 |
| **ΣHCH** | ρ |  |  |  |  |  | 0.97* | 0.382* | 0.28* | 0.42* | 0.409* | -0.098 |
|  | p |  |  |  |  |  | <.0001 | 0.000 | 0.008 | <.0001 | <.0001 | 0.357 |
|  | n |  |  |  |  |  | 90 | 87 | 90 | 90 | 90 | 90 |
| **β-HCH** | ρ |  |  |  |  |  |  | 0.355* | 0.360* | 0.308* | 0.321* | 0.045 |
|  | p |  |  |  |  |  |  | 0.000 | 0.000 | 0.002 | 0.001 | 0.653 |
|  | n |  |  |  |  |  |  | 96 | 103 | 102 | 103 | 103 |
| **ΣCHD** | ρ |  |  |  |  |  |  |  | 0.837* | 0.526* | 0.556* | 0.168 |
|  | p |  |  |  |  |  |  |  | <.0001 | <.0001 | <.0001 | 0.102 |
|  | n |  |  |  |  |  |  |  | 96 | 95 | 96 | 96 |
| **tNCHD** | ρ |  |  |  |  |  |  |  |  | 0.563* | 0.550* | 0.242* |
|  | p |  |  |  |  |  |  |  |  | <.0001 | <.0001 | 0.014 |
|  | n |  |  |  |  |  |  |  |  | 102 | 103 | 103 |
| **ΣDDT** | ρ |  |  |  |  |  |  |  |  |  | 0.950* | 0.016 |
|  | p |  |  |  |  |  |  |  |  |  | <.0001 | 0.872 |
|  | n |  |  |  |  |  |  |  |  |  | 102 | 102 |
| ***p,p’*-DDE** | ρ |  |  |  |  |  |  |  |  |  |  | -0.011 |
|  | p |  |  |  |  |  |  |  |  |  |  | 0.910 |
|  | n |  |  |  |  |  |  |  |  |  |  | 103 |

The p values showing statistical significance (p<0.05) are indicated by ‘*’, and those with marginal significance (p<0.1) are indicated by ‘^’.
