# Supplementary material for: Association between Several Persistent Organic Pollutants and Thyroid Hormone Levels in Cord Blood Serum and Bloodspot of the Newborn Infants of Korea
Source: PLoS One. 2015 May 12;10(5):e0125213. doi: 10.1371/journal.pone.0125213 (PMC4429016; doi:10.1371/journal.pone.0125213)
Supplement: S4 Table — (DOCX) [file pone.0125213.s004.docx]

**Table S4. Results of multivariate analysis with or without maternal thyroid hormone as covariates in the model**

| **Fetal POPs** | **Neonatal thyroid**  **hormone** | **Without maternal thyroid hormone** | |  | **With maternal thyroid hormone** | |
| --- | --- | --- | --- | --- | --- | --- |
|  |  | **β (95% CI)** | **n** |  | **β (95% CI)** | **n** |
| ΣPCB | Cord TT3 | **0.03 (-0.07, 0.07)** | 89 |  | **0.03 ^ (0.00, 0.07)** | **89** |
| *p,p’*-DDE |  | -0.04 ^ (-0.08, 0.00) | 96 |  | **-0.04 ^ (-0.08, 0.00)** | **96** |
| HCB | Cord TT4 | -0.03 * (-0.06, -0.01) | 64 |  | **-0.03 * (-0.06, 0.00)** | **64** |
| BDE99 | Cord TSH | 0.22 * (0.01, 0.42) | 62 |  | **0.21 * (0.00, 0.42)** | **62** |
| ΣCHD |  | 0.15 ^ (0.00, 0.31) | 76 |  | **0.16 ^ (0.00, 0.30)** | **76** |
| ΣPCB | Bloodspot TSH | **-0.16 ^ (-0.33, 0.01)** | **84** |  | -0.17 ^ (-0.35, 0.01) | 81 |
| BDE47 |  | **0.33 * (0.03, 0.62)** | **66** |  | 0.34 * (0.04, 0.63) | 63 |
| β-HCH |  | **-0.27 ^ (-0.57, 0.03)** | **63** |  | -0.25 ^ (-0.55, 0.05) | 63 |
| *p,p’*-DDE |  | **0.21 * (0.03, 0.39)** | **91** |  | 0.21 * (0.06, 0.44) | 88 |
| **Maternal POPs** | **Neonatal thyroid**  **hormone** | **Without maternal thyroid hormone** | |  | **With maternal thyroid hormone** | |
|  |  | **β (95% CI)** | **n** |  | **β (95% CI)** | **n** |
| *β*-HCH | Cord TT3 | -0.04 * (-0.08, 0.00) | 95 |  | **-0.04 * (-0.08, 0.00)** | **95** |
| tNCHD |  | **-0.04 (-0.09, 0.01)** | 95 |  | **-0.05 ^ (-0.10, 0.00)** | **95** |
| ΣCHD | Cord fT4 | -0.04 * (-0.08, -0.01) | 88 |  | **-0.04 * (-0.08, -0.00)** | **88** |
| *p,p’*-DDE |  | -0.02 ^ (-0.04, 0.00) | 95 |  | **-0.02 ^ (-0.04, 0.00)** | **95** |
| ΣCHD | Cord TT4 | -0.06 ^ (-0.12, 0.00) | 88 |  | **-0.06 ^ (-0.12, 0.01)** | **88** |
| *p,p’*-DDE |  | -0.03 ^ (-0.07, 0.01) | 95 |  | **-0.03 ^ (-0.07, 0.00)** | **95** |
| HCB | Cord TSH | 0.10 ^ (0.00, 0.21) | 95 |  | **0.09 ^ (-0.02, 0.19)** | **95** |
| BDE47 | Bloodspot TSH | **0.13 ^ (-0.01, 0.27)** | **90** |  | 0.13 ^ (-0.01, 0.28) | 87 |
| ΣDDT |  | **0.35 * (0.00, 0.69)** | **90** |  | 0.36 * (0.00, 0.71) | 87 |
| *p,p’*-DDE |  | **0.26 * (0.07, 0.45)** | **91** |  | 0.27 * (0.07, 0.46) | 88 |

Signs * and ^ indicate statistical significance of regression parameter at p=0.05, and 0.1, respectively. Red bold values represent difference in significance of association after adjustment of maternal thyroid hormones in the model. ‘fT3’ free T3; ‘TT3’ total T3; ‘fT4’ free T4; ‘TT4’ total T4.
