# Supplementary material for: Association between Several Persistent Organic Pollutants and Thyroid Hormone Levels in Cord Blood Serum and Bloodspot of the Newborn Infants of Korea
Source: PLoS One. 2015 May 12;10(5):e0125213. doi: 10.1371/journal.pone.0125213 (PMC4429016; doi:10.1371/journal.pone.0125213)
Supplement: S1 Text — (DOCX) [file pone.0125213.s005.docx]

**Supporting Materials and Methods (S1 Text)**

**Sample preparation**

The experimental procedures of analysis of OCPs, PCBs and PBDEs in serum were optimized with some modifications of previous studies [1, 2]. In brief, serum samples (2 mL) were fortified with formic acid and Milli-Q water for protein denaturation, after ^13^C-labeled OCPs, PCBs and PBDEs were spiked. The samples were extracted by SPE using Sep-Pak C_18_ SPE cartridge, which was pre-washed with MeOH and conditioned with Milli-Q water. The extracted cartridge was rinsed with Milli-Q water and subsequently dried. A Sep-Pak Plus NH_2_ cartridge, pre-washed with 6 mL of hexane, was connected to the lower end of the C_18_ cartridge. Eight milliliter of hexane was passed through the combined NH_2_-C_18_ cartridges and was collected. After removing C_18_ cartridge, 6 mL of 5% DCM in hexane was passed through NH_2_ cartridge and was combined to a previous fraction. The pooled eluants were cleaned up onto a silica gel/florisil SPE cartridge, using 12mL of 50% DCM in hexane. The purified eluants were concentrated and dissolved in 100μL nonane for instrumental analysis. Measured lipid content of maternal blood and cord blood serum was on average 881 mg/dL and 231 mg/dL in the present population.

**Instrumental analysis and quality control**

A high-resolution gas chromatography interfaced with a high-resolution mass spectrometer (HRGC/HRMS; JMS 800D, JEOL, Tokyo, Japan) was used for the identification and quantification of OCPs, PCBs and PBDEs. Details of instrumental parameters have been reported elsewhere [3, 4]. In brief, OCPs, PCBs and PBDEs were quantified using the isotope dilution method based on relative response factors of individual compounds. The HRMS was operated under positive EI mode, and ions were monitored by selected ion monitoring using molecular ions of target compounds. A DB5-MS (30 m length, 0.25 mm inner diameter, 0.25μm film thickness; J&W Scientific, Palo Alto, CA, USA) was used for the separation of OCPs and PCBs. A DB5-MS (15 m length, 0.25 mm inner diameter, 0.1 μm film thickness; J&W Scientific) was used for the separation of from tri- to heptaBDE congeners.

The recoveries of spiked ^13^C-labeled compounds were 91 ± 10% (average± SD) for OCPs, 62 ± 5.5% for PCBs and 87 ± 13% for PBDEs. Solvents injected before and after the injection of standards showed negligible contamination or carryover. Procedural blanks (*n* = 10) were processed with every set of 15 serum samples to check laboratory contamination. Blanks did not contain quantifiable amounts of target contaminants.

**References**

[1] Dmitrovic J, Chan SC, Chan SH (2002) Analysis of pesticides and PCB congeners in serum by GC/MS with SPE sample cleanup. Toxicol Lett 134:253-8.

[2] Kang JH, Park H, Chang YS, Choi JW (2008) Distribution of organochlorine pesticides (OCPs) and polychlorinated biphenyls (PCBs) in human serum from urban areas in Korea. Chemosphere 73:1625-31.

[3] Moon HB, Kannan K, Lee SJ, Choi M (2007) Polybrominated diphenyl ethers (PBDEs) in sediment and bivalves from Korean coastal waters. Chemosphere 66:243-51.

[4] Moon HB, Kim HS, Choi M, Yu J, Choi HG (2009) Human health risk of polychlorinated biphenyls and organochlorine pesticides resulting from seafood consumption in South Korea, 2005-2007. Food Chem Toxicol 47:1819-25.
